# Combination therapy of orally administered glycyrrhizin and UVB improved active-stage generalized vitiligo

**DOI:** 10.1590/1414-431X20165354

**Published:** 2016-07-25

**Authors:** K.H. Mou, D. Han, W.L. Liu, P. Li

**Affiliations:** 1 Department of Dermatology, The First Affiliated Hospital, Xi'an Jiaotong University, Xi'an, China; 2 Center for Translational Medicine, The First Affiliated Hospital, Xi'an Jiaotong University, Xi'an, China

**Keywords:** Glycyrrhizin, UVB, Generalized vitiligo, Disease stage, Active stage

## Abstract

Glycyrrhizin has been used clinically for several years due to its beneficial effect on immunoglobulin E (IgE)-induced allergic diseases, alopecia areata and psoriasis. In this study, glycyrrhizin, ultraviolet B light (UVB) or a combination of both were used to treat active-stage generalized vitiligo. One hundred and forty-four patients between the ages of 3 and 48 years were divided into three groups: group A received oral compound glycyrrhizin (OCG); group B received UVB applications twice weekly, and group C received OCG+UVB. Follow-ups were performed at 2, 4, and 6 months after the treatment was initiated. The Vitiligo Area Scoring Index (VASI) and the Vitiligo Disease Activity (VIDA) instrument were used to assess the affected body surface, at each follow-up. Results showed that 77.1, 75.0 and 87.5% in groups A, B and C, respectively, presented repigmentation of lesions. Responsiveness to therapy seemed to be associated with lesion location and patient compliance. Adverse events were limited and transient. This study showed that, although the three treatment protocols had positive results, OCG and UVB combination therapy was the most effective and led to improvement in disease stage from active to stable.

## Introduction

Vitiligo, a common idiopathic acquired depigmentation disorder, occurs mostly in young people, who are typically concerned about their appearance. It may be associated with significant psychological trauma that has lasting effects on the person's self-esteem. The causes of vitiligo remain unknown. However, current vitiligo treatment options are considered to be suboptimal because they are not always effective and some therapies are limited to certain types of vitiligo.

Skin transplantation is an effective way to locally treat inactive vitiligo but it is not suitable for generalized, active-stage vitiligo (1). Several clinical trials have been conducted to explore treatments of generalized, active-stage vitiligo. The studies indicate that nearly all treatment options are not completely effective (2). Narrow-band ultraviolet B light (UVB) therapy and oral cortisone or methotrexate have been reported to be effective treatment options in some patients with this condition (3). However, the side effects cannot be neglected in certain patients or in children, and some reports show that there were high rates of relapse after cortisone therapy (4).

Glycyrrhizin and its aglycone, glycyrrhetinic acid, have a wide range of pharmacological actions including anti-inflammatory, anti-allergic, anti-viral, anti-carcinogenic, anti-thrombin and anti-immune-mediated cytotoxicity. It has been reported that glycyrrhizin can relieve immunoglobulin E (IgE)-induced allergic diseases such as dermatitis (5). In children, oral compound glycyrrhizin (OCG) tablets and total glucosides of paeony capsules can effectively treat severe alopecia areata because of their anti-inflammation and immunoregulation functions (6). Methotrexate and compound glycyrrhizin can be an effective alternative therapy in the treatment of erythrodermic psoriasis with bullous pemphigoid (7). Glycyrrhizin can also effectively enhance the curative effects of acitretin in the treatment of psoriasis (8), and increase tyrosinase gene expression and protein content and tyrosinase-related protein 2, which is critical for melanin synthesis (9). OCG therapy side effects are mild, and the most common ones are edema or low-serum potassium levels, which can be easily managed. The advantage of OCG is that it can be used to control rapidly progressing lesions in patients who need glucocorticoids, or even methotrexate, but cannot tolerate the side effects of these drugs (10).

Because the effects of OCG are similar to cortisone, its many therapeutic functions (11) and its mild side effects, we conducted an open trial to study the efficacy of OCG in patients with generalized, active-stage vitiligo. In this study, we compared the effects of treatment with OCG and UVB combination therapy with UVB and OCG alone.

## Material and Methods

This study was conducted from January 2011 to March 2012 in the Department of Dermatology and Venereology, the First Affiliated Hospital, Xi'an Jiaotong University, in Shaanxi Province, China. The study protocol was approved by the hospital's Ethics Committee.

### Study participants

Patients who presented at our hospital seeking treatment for vitiligo were approached for enrollment in the study. All patients had generalized, active-stage vitiligo, with a minimal depigmentation of 6.8%. The Vitiligo Area Scoring Index (VASI) was used to assess the affected body surface, and patients with a Vitiligo Disease Activity (VIDA) score greater than one and without local or systemic immunosuppressive treatment for vitiligo in the past 6 months were selected. Patients with a history of previous side effects or phototoxic reactions related to photo(chemo)therapy, a history of photosensitivity or photomediated disorders, skin type I (according to Fitzpatrick classification I–VI), concomitant radiotherapy, chemotherapy or immunosuppressive therapy, and claustrophobia were excluded from the study. Written informed consent was obtained from each patient before any study-related investigation or intervention. One hundred and forty-four patients between the ages of 3 and 48 years were allocated into 3 groups of 48 each: group A, treated with OCG alone; group B, treated with UVB twice weekly; and group C treated with OCG and UVB, for 6 months. Follow-ups were scheduled every 2 months and were continued for up to 6 months.

Before the study, a physical examination and routine laboratory tests were performed to exclude some systemic diseases such as hypertension, edema, heart and kidney diseases. During the study, patients were not allowed to take any other systemic or topical medications.

### Study design

The study was an open-label, randomized trial. There were no significant differences (in age, gender, disease activity) among the three treatment groups (P>0.05; Table 1). Subjects were screened during a preliminary visit, approximately 1–2 weeks before starting treatment. Follow-ups were performed at 2, 4 and 6 months after treatment.

**Table:**
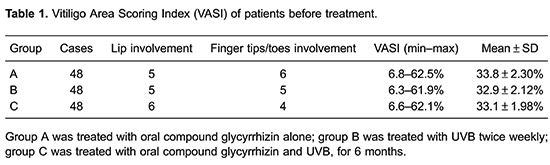

### Treatment period

OCG tablets are produced in Japan by Minophagen Pharmaceutical Co. Ltd. (12). They consist of 20 mg glycyrrhizin, 25 mg aminoacetic acid and 25 mg methionine per tablet. The tablets were administered orally after a meal to group A. The dosage was based on the patient's weight and age. Patients weighing less than 60 kg or younger than 12 years of age received one tablet three times per day. Patients with 60 kg or more, or over 12 years of age received two tablets three times per day. This therapy was used for the entire trial period. Patients were withdrawn from the trial if they felt significant fatigue or if edema or high blood pressure developed.

Twelve narrow-band fluorescent tubes (Philips TL 100W/01, Netherlands), with a spectrum between 310 and 315 nm and a maximum wavelength of 311 nm, were installed in a Waldmann UV-1000 cabinet (Germany). The radiance in the UVB cabinets was checked routinely using a UVB detector. The average radiance was 10.0 mW/cm^2^.

Narrow-band UVB therapy was administered twice weekly to group B, with at least 1 day between each treatment. The initial dose was 0.25 J/cm^2^, independent of the skin type. The dose was increased by 20% with each treatment. The optimal constant dose was achieved when minimal erythema occurred in the lesions. During treatment, the eyes were protected by UV-blocking goggles. If significant depigmentation was present in the eyelids and patients insisted on treating these areas, they were told to keep their eyes shut during treatment. All patients kept their underwear on to shield the genitals from narrow-band UVB exposure. The patients were advised to protect their skin against excessive exposure to natural sunlight, especially between 11:00 am and 3:00 pm on sunny days, on both treatment and non-treatment days. A sunscreen with a high protection factor (SPF 25 or higher) was applied daily on sun-exposed areas.

The OCG therapy combined with narrow-band UVB therapy was administered to group C. The OCG tablet was taken orally every day for 6 months. The dosage and precautions were the same, as mentioned above. For narrow-band UVB therapy, treatment intervals and precaution measures were the same as mentioned above.

### Analysis and evaluation of treatment effects

A survey table was constructed before the trial. It included information on gender, age, hereditary history and history of vitiligo, treatment, photosensitivity and other diseases. A blank space was left for VASI and VIDA for follow-up data.

A full-body standard pose photograph of the front and back was taken of each patient. The photographs were taken before treatment and at each visit. The VASI and VIDA scores were estimated by two independent observers also before treatment and at every visit, as described above. The VASI and VIDA are scoring systems based on the observer's subjective impression of the patient's present disease activity within the indicated time periods, as follows: active in the past 6 weeks (score +4), active in the past 3 months (score +3), active in the past 6 months (score +2), active in the past year (score +1), stable for at least 1 year (score 0); and stable for at least 1 year with spontaneous repigmentation (score –1). The term "active" is defined as the expansion of existing lesions or the appearance of new lesions. "Stable" refers to the condition when these symptoms are not present. The phototherapy notes were reviewed for information about the duration of treatment, the total number of applications, the cumulative narrow-band UVB dose and adverse effects.

### Patient's quality of life questionnaire

A Dermatology Life Quality Index (DLQI) table was used to evaluate the psychosocial impact of the disease and treatment. Permission to use the DLQI was provided by Dr. Dan Han from our department. The initial version was validated by two translations performed by two independent translators. The final version was assembled using the translations and revisions proposed by Dr. Wenli Liu.

The DLQI involves 10 questions regarding the patient's subjective perception of the effects of the disease on daily life. Each question was evaluated using a 4-point scale: 0, no effect; 1, mild effect; 2, significant effect, and 3, very pronounced effect. All patients were asked to complete the DLQI (with the help of their parents, if the patient was a child) at two time points: before and after the termination of therapy.

### Statistical analysis

Statistical analysis was performed using the chi-square test, the paired or unpaired two-tailed Student's *t*-test, or the Fisher exact test, depending on the type of data set involved. The significance level was set at P<0.05. Analyses were performed using the SPSS software (IBM, USA).

## Results

### Changes in vitiligo disease activity score before and after treatment

There was no statistically significant difference among the three groups in vitiligo activity before treatment (P>0.05), which meant the groups were comparable. After 2, 4 and 6 months of treatment, the total VIDA score of group A fell continuously to 102 at 2 months, 46 at 4 months, and 32 at 6 months. The total VIDA scores of group B were 98, 50 and 38 at 2, 4 and 6 months, respectively. Group C scores were 82, 42 and 21, for the same time points. The VIDA score in all groups decreased during treatment, which demonstrates that both OCG and UVB were effective. In the 2nd and 6th months of treatment, the scores of group C were significantly lower than groups A and B (P<0.05), which demonstrates that the treatment for group C was more effective in the improvement of vitiligo compared with groups A and B (Table 2).

**Table:**
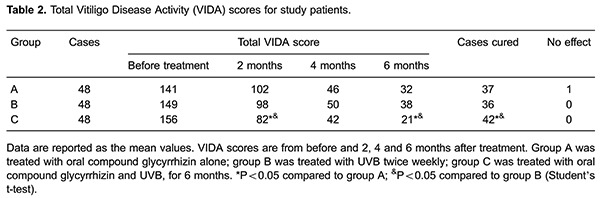

### Effectiveness rate

An overall repigmentation rate of 77.1% (37/48) was observed in group A, 75.0% (36/48) in group B and 87.5% (42/48) in group C. The differences between groups A and C, and B and C were significant (P<0.05), which showed that OCG plus UVB was superior to OCG or UVB alone in the treatment of generalized, active-stage vitiligo (Table 2).

### Safety results

One patient in group A complained of facial edema after oral OCG therapy, which lasted for about 8 days, and after prudent continuous use, the symptoms disappeared. Facial edema also occurred in one patient in group C, but it slowly disappeared and had no effect on the course of treatment. No other severe conditions such as hypertension, low blood potassium levels or cardiac symptoms were observed. A 12-year-old girl showed no treatment effect after completing the OCG therapy. Thereafter, she received approximately 5 months of narrow-band UVB therapy and attained satisfactory repigmentation (Table 2).

All patients in groups B and C finished the entire course of narrow-band UVB therapy, and all exhibited a reaction to the therapies of varying degrees (Table 2); there were no severe side effects and no withdrawal symptoms.

### Patient's quality of life questionnaire (DLQI)

The DLQI scores were high before treatment: 4.8±4.5 for group A, 6.3±4.8 for group B, and 5.6±3.2 for group C (P>0.05). After treatment, scores decreased markedly: 2.9±2.6 for group A, 3.1±2.4 for group B, and 1.8±1.5 for group C (P<0.001, compared to before treatment), as shown in Table 3.

**Table:**
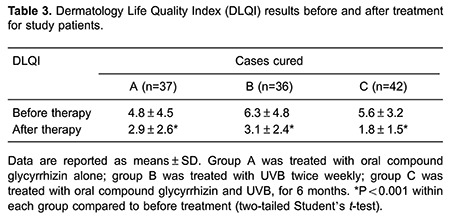

## Discussion

Several treatment modalities have been used to treat generalized vitiligo in the active stage (13). Oral trimethylpsoralen plus sunlight showed variable results in several independent studies (14). Psoralens must be used with caution because of their phototoxic properties; other known side effects of this class of drugs include nausea, pruritus and increased contrast between the lesion and normally pigmented skin. In general, oral psoralen plus UVA (PUVA) is not recommended for children under 12 years of age. It is known that repigmentation by PUVA is a long and tedious process that can require months or years of treatment. Both patients and dermatologists are concerned with its long-term use and dermatologists are reluctant to prescribe phototherapy to patients because of practical difficulties (15). L-Phenylalanine plus UVA have also been reported to yield good results in patients with slowly spreading vitiligo (16), but results have not been confirmed. The administration of systemic steroids in children was reported to alleviate vitiligo in some cases, especially the rapidly progressing type, but the risk of suppression of the adrenal cortex should not be underestimated. Although steroids can adequately control the condition, treatment interruption is difficult and vitiligo lesions can recur easily after interruption. Several studies suggest using methotrexate to control active vitiligo (17), but the side effects of this drug are significant (myelosuppression and hepatotoxicity) and it is not well tolerated by all patients. Topical steroids or topical tacrolimus are sometimes used (18), with variable results, to treat limited areas in patients with extensive vitiligo. Skin transplantation can be effective, but it is a passive treatment and is not suitable for unstable vitiligo (19). Therefore, there is still no effective and safe therapy available to treat patients who do not satisfactorily respond to these therapies, especially those who are in the active stage (20).

To date, researchers believe that UVB treatment (or even treatment with high-energy excimer laser, 308 nm) is an effective method to promote pigment recovery, and that its side effects (such as skin cancer, photoaging) are less frequent than when PUVB is used. Therefore, it has been suggested that UVB may replace PUVB. Clinically, not every patient responds satisfactorily to UVB, so some researchers have suggested there is a need for some type of combination therapy or an individualized approach to treatment (21).

OCG has been used in several dermatologic disorders for years. It is commonly used to treat mild to moderate alopecia areata, because it effectively inhibits CD4^+^ and CD8^+^ cells and their cytokine generation (22). A 68-year-old male who had erythrodermic psoriasis with bullous pemphigoid was successfully treated using a combination of methotrexate and compound glycyrrhizin (7). It has been reported that total glucosides of paeony capsule (TGPC) plus OCG is more effective than OCG alone in the treatment of severe alopecia areata in children (6). Another study suggested that the combined use of compound glycyrrhizin and acitretin can produce a synergistic effect by antagonizing Th17 cell-related mechanisms for psoriasis treatments (8).

The pathogenesis of vitiligo has not been clear so far, but studies have demonstrated that inflammation is present in the dermis. It has showed that altered cellular immunity is present in vitiligo, in addition to and perhaps in combination with a humoral immune response (23). The OCG can inhibit inflammatory actions and regulate T cell activation. It acts like corticosteroids with nearly no side effects, such as hairiness, sanguineous temperament, immunity inhibition and osteoporosis. Therefore, it is reasonable to assume that OCG is effective in vitiligo treatment.

The results of this study indicate that OCG plus narrow-band UVB or the 308-nm excimer laser therapy can represent a valuable and safe option for treating vitiligo in the active stage. The treatment may result in improved cosmetic appearance and psychosocial functioning of vitiligo patients. OCG can help make the disease more stable (2) and narrow-band UVB treatment can promote quick pigment recovery (24). The mechanism of narrow-band UVB therapy could be by stimulating melanocytic reserves in the hair sheaths, because in our study, repigmentation occurred in a perifollicular pattern and was not observed in lesions with white amelanotic hairs.

In the present study, when OCG combined with narrow-band UVB was administered, the percentage of patients achieving overall repigmentation was 87.5%, which are promising results for active-stage vitiligo patients. In group A, there was a 77.1% overall repigmentation rate at the last visit, indicating that treatment with OCG alone can also result in satisfactory results in vitiligo patients. Those patients who are allergic to, or refuse to receive UVB treatment, can opt for OCG only.

Although this was an open study and did not have a negative control group, most of the observed repigmentation could be attributed to OCG plus narrow-band UVB therapy. The majority of patients (135/144, 93.75%) had not experienced previous "spontaneous" repigmentation. Moreover, spontaneous repigmentation is usually partial and has never been reported to include more than 75% of the lesions. In addition, the patients were repeatedly instructed to protect their skin from extra sun rays and to apply sun block lotion, as previously described (25). Finally, most of the patients who did not respond to previous treatments showed excellent repigmentation after therapy with OCG and narrow-band UVB.

Some skin areas responded better to therapy than others. The face and neck showed the best results, whereas the trunk and proximal extremities exhibited moderate repigmentation. In contrast, the acral sites (fingers, feet), and areas of bony prominences and with lower hair density (wrists, ankles and joints), showed little repigmentation. This finding may be related to blood circulation, as areas that were more proximal to the heart recovered more quickly.

DLQI scores revealed that vitiligo has a negative impact on patients' quality of life. With the repigmentation of white patches, especially those on the face and other exposed areas, the DLQI scores significantly reduced.

There are some limitations to our study, such as the small sample size and relatively short investigative period. Therefore, a multicenter, randomized controlled trial is still needed to substantiate our findings. Properly designed follow-up studies should investigate the permanency of OCG plus narrow-band UVB therapy-induced repigmentation in acute-stage vitiligo.
